# Single-Nucleosome Mapping of Histone Modifications in *S. cerevisiae*

**DOI:** 10.1371/journal.pbio.0030328

**Published:** 2005-08-30

**Authors:** Chih Long Liu, Tommy Kaplan, Minkyu Kim, Stephen Buratowski, Stuart L Schreiber, Nir Friedman, Oliver J Rando

**Affiliations:** **1**Bauer Center for Genomics Research, Harvard University, Cambridge, Massachusetts, United States of America,; **2**Department of Chemistry and Chemical Biology, Harvard University, Cambridge, Massachusetts, United States of America,; **3**School of Computer Science and Engineering, The Hebrew University, Jerusalem, Israel,; **4**Department of Molecular Genetics and Biotechnology, The Hebrew University, Jerusalem, Israel,; **5**Department of Biological Chemistry and Molecular Pharmacology, Harvard University, Boston, Massachusetts, United States of America; Adolf Butenandt InstituteGermany

## Abstract

Covalent modification of histone proteins plays a role in virtually every process on eukaryotic DNA, from transcription to DNA repair. Many different residues can be covalently modified, and it has been suggested that these modifications occur in a great number of independent, meaningful combinations. Published low-resolution microarray studies on the combinatorial complexity of histone modification patterns suffer from confounding effects caused by the averaging of modification levels over multiple nucleosomes. To overcome this problem, we used a high-resolution tiled microarray with single-nucleosome resolution to investigate the occurrence of combinations of 12 histone modifications on thousands of nucleosomes in actively growing *S. cerevisiae*. We found that histone modifications do not occur independently; there are roughly two groups of co-occurring modifications. One group of lysine acetylations shows a sharply defined domain of two hypo-acetylated nucleosomes, adjacent to the transcriptional start site, whose occurrence does not correlate with transcription levels. The other group consists of modifications occurring in gradients through the coding regions of genes in a pattern associated with transcription. We found no evidence for a deterministic code of many discrete states, but instead we saw blended, continuous patterns that distinguish nucleosomes at one location (e.g., promoter nucleosomes) from those at another location (e.g., over the 3′ ends of coding regions). These results are consistent with the idea of a simple, redundant histone code, in which multiple modifications share the same role.

## Abstract 2

High-resolution microarrays were used to investigate 12 histone modifications across thousands of yeast nucelosomes in vivo. Two main groups co-occurred, consistent with the redundant histone code hypothesis.

## Introduction

Nucleosomes play many roles in transcriptional regulation, ranging from repression through occlusion of binding sites for transcription factors [1], to activation through spatial juxtaposition of transcription factor-binding sites [2]. There are two main ways in which cells modulate nucleosomal influences on gene expression. One way is through chromatin remodelling, using the energy of adenosine triphosphate hydrolysis to modulate nucleosomal structure, often resulting in changed nucleosomal location [3]. Alternatively, covalent histone modifications have many effects on transcription. Histone proteins have highly conserved tails, which are subject to multiple types of covalent modification, including acetylation, methylation, phosphorylation, ubiquitination, sumoylation, and adenosine-diphosphate ribosylation [4–9].

Histone acetylation has been the subject of decades of research, whereas histone methylation has come under intense scrutiny more recently. Lysine acetylation neutralizes lysine's positive charge, and can influence gene expression in at least two ways. Firstly, charge neutralization can affect contacts between the positively charged histone tail and negatively charged neighbouring molecules, such as adjacent linker DNA [10], or acidic patches on histones in nucleosomes [11]. Alternatively, acetyl-lysine is bound by the bromodomain, a protein domain found in many transcriptional regulators; thus, acetylation might affect recruitment of protein complexes [12]. Histone acetylation is rapidly reversible, and acetyl groups turn over rapidly in vivo, with half-lives on the order of minutes [13], allowing for rapid gene expression changes in response to signals [14]. Acetylation of histone lysines has been associated with both transcriptional activation and transcriptional repression [15–17]. The outcome of acetylation depends on which lysine is acetylated and the location of the modified nucleosome. A recent genome-scale study of histone acetylation in yeast revealed a complicated relationship between histone modification and transcriptional output [18].

Histone methylation has been best characterized by histone 3-lysine 4 (H3K4), wherein methylation is associated with active transcription in multiple organisms, ranging from *Saccharomyces cerevisiae* to mammals. Lysine can be mono-, di-, or tri-methylated, and none of these methylation states will alter lysine's positive charge (under conditions of standard lysine pKa and physiological pH). As a result, it is unlikely that charge–charge interactions are modulated by methylation, which appears instead to affect cellular processes through binding of methyl-lysine–binding proteins. Indeed, methyl-lysine is bound by at least one domain type—the chromodomain [19,20]. In contrast to histone acetylation, histone methylation is long-lived. Although a histone-lysine demethylase (termed LSD1) was recently identified in metazoans. *S. cerevisiae* does not have a homolog of this protein. Even in metazoans, the proposed enzymatic mechanism allows for demethylation of mono- and di-methylated lysine, but not of tri-methylated lysine [21]. Whether or not enzymatic demethylation of tri-methyl-lysine occurs, and whatever other mechanisms allow for replacement of tri-methylated histones (such as histone replacement—[22]), in yeast, H3K4 tri-methylation is associated with active transcription. The histone tri-methylation persists for over an hour after transcription ceases, providing a memory of recent transcription [23].

The discovery of multiple modification types and modified residues suggested that different combinations of histone modifications might lead to distinctive transcriptional outcomes. According to the “histone code” hypothesis, “distinct histone modifications, on one or more tails, act sequentially or in combination to form a ‘histone code' that is read by other proteins to bring about distinct downstream events” [6].

This hypothesis has been the subject of much debate, much of it concerning the requirements for histone modifications to form a “code” [4–9]. In this study, we focused on the combinatorial complexity of histone modification patterns. Insights into this complexity require an understanding of which combinations of modifications occur in vivo, and the functional consequences of these combinations. Mutagenesis of histone tails has demonstrated that not all combinations of histone modifications lead to distinct transcriptional states [24]. In addition, genome-wide localization studies of histone modifications in yeast, flies, and mammals have demonstrated that not all possible histone-modification patterns occur in vivo [18,25,26].

A major confounding effect in the interpretation of previous genome-wide studies of histone modifications in vivo is the low resolution of the measurements (~500–1,000 base pairs [bp]) relative to the size of the nucleosome (~146 bp). Thus, the measured ratio for a given spot represents an aggregate that is actually an average of information from several nucleosomes, which complicates analysis. Furthermore, in some studies, acetylation patterns at intergenic and coding regions were measured using different microarrays, precluding a common reference point. Finally, whole genomic DNA has typically been used as the reference DNA in these microarray studies, thereby confounding the measurements of histone modification with underlying variation in nucleosome density [27,28].

To overcome these limitations, we made use of a recently developed, high-density oligonucleotide microarray with ∼20-bp resolution. We recently used this microarray to map nucleosome positions across almost half a megabase of the yeast genome [29]. In this study, we use this microarray to measure the levels of 12 different histone modifications in individual nucleosomes. We find that modifications do not occur independently of each other and that a small number of distinct combinations occur in vivo. Different modification patterns are enriched at specific locations in gene or promoter regions, and these patterns are predictive of the transcription level of the underlying gene. Sharp transitions in histone modifications mostly occur near the transcription start site (TSS). Together these results provide a simpler view of histone modification, and suggest that there is little combinatorial information encoded in the histone tails.

## Results

### High-Resolution Measurement of Histone Modifications Using Tiled Microarrays

**Figure 1:**
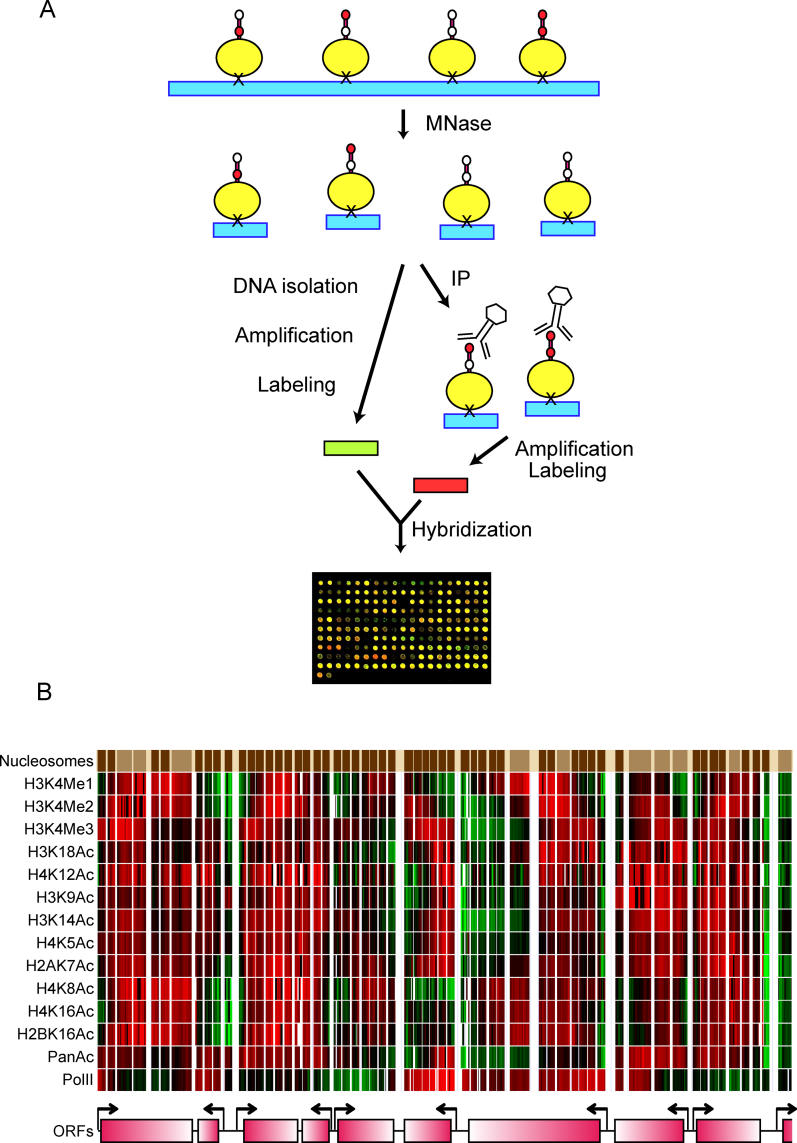
Overview (A) Nucleosomes are first cross-linked to DNA using formaldehyde. Cross-linked chromatin is digested to mononucleosomes with micrococcal nuclease. Mononucleosomal digests are immunoprecipitated using an antibody specific to a particular histone modification, and immunoprecipitated DNA is isolated and labelled with Cy5. DNA is also isolated from the same nuclease titration step prior to immunoprecipitation, labelled with Cy3, and mixed with Cy5-labeled immunoprecipitated DNA. Labelled DNA is then hybridized to a tiled microarray covering half a megabase of yeast genome. (B) Example of raw data. Data are shown for all modifications tested, along with PolII data. Red (green) indicates enrichment (depletion), while grey indicates missing data. Data from probes found in linker regions are not shown. Each row represents median data from multiple replicates with one antibody, as indicated (PanAc refers to a nonspecific antibody to acetyl-lysine, which we used to measure bulk acetylation). “Nucleosomes” shows positions of nucleosomes previously described [29], with dark brown for well-positioned nucleosomes, very light brown for linkers, and intermediate brown for delocalized nucleosomes. “ORFs” shows locations of annotated genes. Data shown are for Chromosome III coordinates 58,900 to 72,100.

Chromatin immunoprecipitation (ChIP) using modification-specific antibodies [30,31] was used to map histone modifications in actively growing yeast cultures. We used a standard ChIP protocol, with one major modification (Figure 1A). In our protocol, formaldehyde-fixed yeast were lysed gently by spheroplasting and osmotic lysis rather than by glass beads, and DNA was digested to mononucleosomes using micrococcal nuclease (rather than sheared to ~500 bp by sonication) (Figure S1). This allowed us to map modifications at nucleosomal resolution. We used antibodies specific to 12 individual modifications, including mono-, di-, and tri-methylation of histone H3K4, as well as acetylation of various lysines on all four histones. Immunoprecipitated DNA was isolated, linearly amplified [32], and labelled with Cy5 fluorescent dye, while mononucleosomal DNA treated under identical conditions was used as the “input” and labelled with Cy3. This choice of input served to control for nucleosomal occupancy differences (to prevent highly modified, low-occupancy nucleosomes from appearing to be poorly modified nucleosomes), as it has been shown that nucleosomes are not always present in every cell in a population [33,34]. Mixtures were hybridized to a tiled microarray covering half a megabase of yeast genomic sequence, including almost all of Chromosome III as well as 230 additional 1-kb promoter regions [29]. This represents approximately 4% of the yeast genome, and includes a total of 356 promoter regions. Finally, to measure active transcription (while avoiding effects of mRNA instability that influence mRNA abundance measurements), we also immunoprecipitated DNA associated with RNA polymerase II (this DNA was sheared by sonication rather than cut with micrococcal nuclease) [35].

### A Chromosomal View of Histone Modifications

The resulting data provide a rich view of histone modification over half a megabase of yeast sequence, demonstrating several prominent features (Figure 1B shows a sample stretch). First, histone modifications generally occur in broad domains, and there are few examples of nucleosomes whose modification pattern was significantly different from that of their adjacent nucleosomes. This was not due to limitations in the experimental technique, as we did find multiple examples of punctate nucleosomes that occurred in expected locations (see below). Second, modifications were generally homogeneous for all the probes within a given nucleosome. Third, correlations could be observed between a nucleosome's position relative to coding regions and its modification pattern. For example, most of the open reading frames shown in Figure 1B exhibit a striking pattern of histone H3K4 methylation, with tri-methylation occurring at the 5′ end of the coding region, shifting to di-methylation, and then to mono-methylation. This pattern is clear over most expressed open reading frames on Chromosome III, and is consistent with reports that Set1 association with RNA polymerase is responsible for methylation of this lysine [23,36]. Finally, we noticed broad domains of low acetylation occurring over heterochromatic regions on our array—subtelomeric sequences and the silent mating type loci [37] (Figure S2).

### Coupling of Modifications to Organization of Transcriptional Units

**Figure 2:**
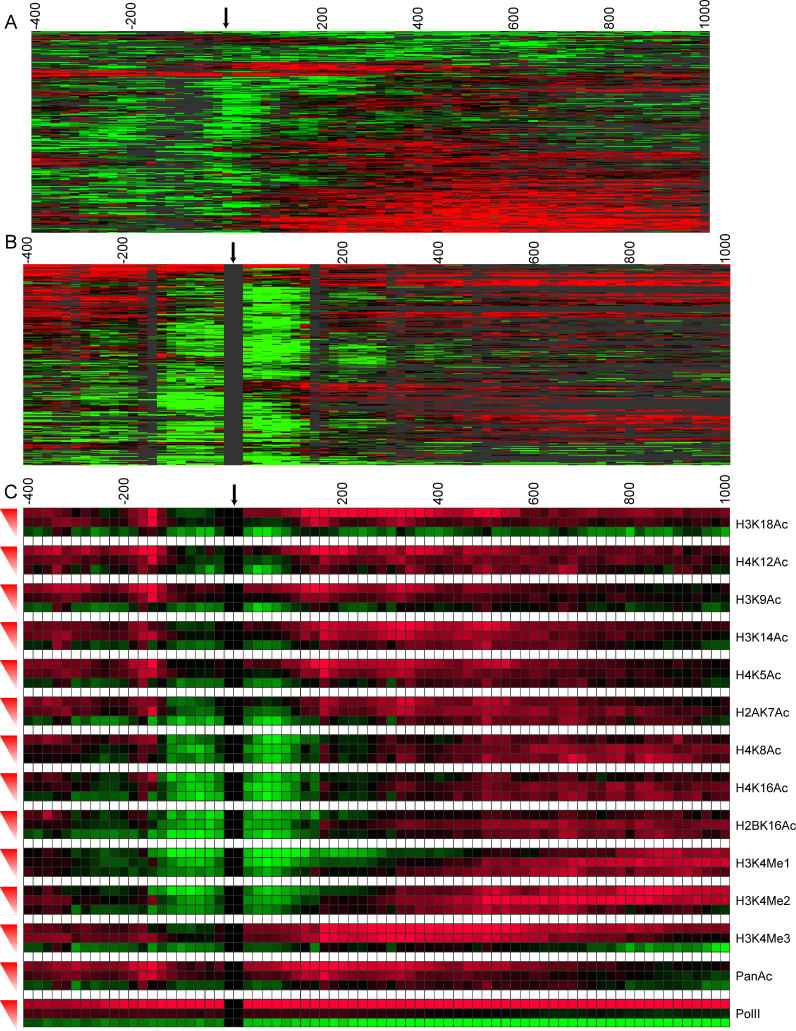
Broad Patterns of Histone Modifications (A) H4K16Ac aligned by ATG. In this representation, the horizontal axis represents location relative to the downstream gene's start codon, and each horizontal line represents one PolII-driven gene. Each cell in the resulting matrix corresponds to the acetylation level at a given microarray probe for one tail position. Red (green) cells mark hyper-acetylated (hypo-acetylated) probes. Non-nucleosomal probes are blackened. We clustered the promoters using a probabilistic agglomerative clustering algorithm (see Materials and Methods). Arrow indicates annotated ATG. (B) H4K16 aligned by transcriptional start site, as in (A), except that arrow indicates TSS (identified in [29]) and data before and after the TSS are aligned by the first nucleosome in that direction. (C) Relationship of histone modification patterns to transcription level. Genes were split into three groups based on PolII enrichment, and averaged data for these groups are shown as indicated, aligned as in (B). Transcription level is indicated by red triangles to the left of each set of three rows.

To analyze the relationship of different modifications to the underlying sequence, we aligned all genes (and their promoters) by their start codon. For example, Figure 2A shows data for histone H4K16 acetylation on aligned genes that were clustered to highlight patterns (see Materials and Methods). Clearly notable in this representation is a hypo-acetylated domain adjacent to most start codons. We have recently discovered that TSSs are found in long nucleosome-free regions [29]. By aligning genes by the location of the first nucleosome following the TSS, a clear domain of two hypo-acetylated nucleosomes can be observed at most PolII promoters (Figure 2B). This alignment, therefore, provides a highly informative view of the relationship of histone modifications to the underlying structure of the genome (see Figure S3 for the remaining modifications).

To explore the relationship of these modifications to transcription, we separated genes into “bins” of varying transcriptional activity (see Materials and Methods) and averaged the enrichment data for all aligned genes in each bin (Figures 2C and S4). Several previously identified features of yeast chromatin are apparent. First, histone H3K4 methylation enrichment correlates with transcription levels, and occurs in a 5′ to 3′ gradient (as also seen in Figure 1B) with tri-methyl enrichment at the 5′ end of genes, shifting to di-methyl and then mono-methyl. Histone H3K4 is methylated by Set1, which is associated with elongating RNA polymerase [23,36], and, as noted above, this gradient presumably reflects the kinetics of dissociation of Set1 from the polymerase, convoluted with the ensemble-average location of polymerase. Second, we reproduced previous observations that histone H3K9/K14 acetylation is enriched over the 5′ ends of coding regions [26,38].

Figure 2C also reveals novel locations of particular histone modification patterns. In particular, the two-nucleosome hypo-acetylation domain described above for H4K16 acetylation is surprisingly general, and a nearly identical pattern is also seen for acetylation of H4K8 and of H2B K16 (Figures S3 and 2C). This hypo-acetyl domain does not correlate with transcription levels (as measured by either PolII occupancy or by mRNA abundance [Figures 2C and S4]). Also, the acetylation of these residues at the middle and 3′ ends of coding regions is either uncorrelated (H2BK16) or anticorrelated (H4K8 and K16) with transcription (Figure 2C). We will therefore refer to this group of modifications as the *transcription-independent* modifications, for convenience (and to emphasize the stereotyped promoter-deacetyl domain). A two-nucleosome hypo-acetylation domain is also present at a smaller subset of promoters for the remaining acetylation states, and is generally found preferentially in poorly expressed genes (Figures S3 and 2C). However, the acetylation of these lysines is found at the 5′ end of coding regions, whereas acetylation of the transcription-independent group is largely excluded from 5′ coding regions. We will refer to this 5′-directed group of modifications as the *transcription-dependent* modifications. Acetylation of H2A K7 is an interesting case, as its pattern appears to be a mixture of the two types of patterns described. However, we have recently found that the H2A isoform Htz1 is enriched in a pattern that dramatically parallels the hypo-acetylation domain observed for the transcription-independent modifications (unpublished data), so H2A is expected to be depleted in this region. This, coupled with the 5′-enrichment of acetylation seen for H2A K7, in highly transcribed genes, leads us to include this modification in the transcription-dependent group.

### Low Dimensionality of Nucleosome Modification Patterns

**Figure 3:**
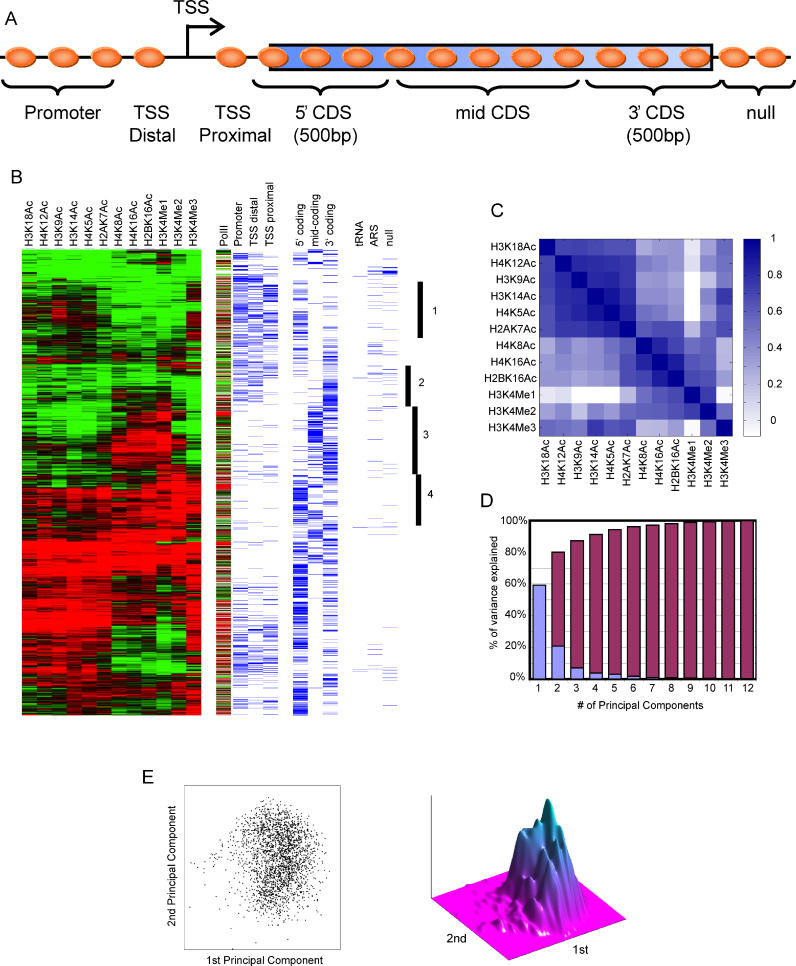
Nucleosome Modification Patterns (A) Schematic of annotation scheme for nucleosomes based on their position relative to transcribed units. Intergenic nucleosomes were assigned to the following categories: promoter region (anything upstream of a coding region), nucleosome immediately upstream to the TSS (“distal”), and the nucleosome immediately downstream of the TSS (“proximal”). Transcribed regions were separated into 5′, middle, and 3′ CDSs. Finally, to capture features of chromatin not associated with PolII genes, we independently classified nucleosomes associated with ARS sequences, tRNA genes, and Null (any other intergenic region). (B) Hierarchical clustering of 2,288 nucleosomes. Left panel: each row corresponds to a single nucleosome, and each column to a particular modification. Red (green) denotes hyper-acetylation (hypo-acetylation) in the first nine columns and relative level of methylation in the last three columns. Rows are sorted according to the dendogram built during clustering. PolII shows the PolII occupancy of the gene associated with the nucleosome in question. Right panel: each row corresponds to a nucleosome (matching the left panel), and each column corresponds to an annotation of the nucleosome according to the scheme of (A). A blue cell denotes a positive annotation of the nucleosome with the appropriate column label. Numbers indicate examples of clusters, as follows: (1) nucleosomes enriched for H3K9Ac, H3K14Ac, and H3K4Me3 that are mostly upstream of transcribed regions; (2) strongly hypo-acetylated nucleosomes, mostly at upstream regions or 3′ of coding regions; (3) nucleosomes acetylated at H4K8 and K16, and H2B K16 that are almost exclusively at the middle and 3′-ends of coding regions; and (4) hyper-acetylated and methylated nucleosomes that are mostly found at the 5′-end of coding regions. (C) The Pearson correlations of the 12 modification levels between different probes show that there are two tightly correlated groups of acetylations at specific residues. The first group consists of H2A K7; H3K9, K14, and K18; and H4K5 and K12. The second group consists of H2B K16; and H4K8 and K16. Mono- and di-methylation of H3K4 are correlated with the second group, while tri-methylation of H3K4 is correlated with the first group. (D) The percent of variance captured by using different number of components. The *x*-axis denotes the number of components, and the *y*-axis denotes the percent of the variance in the data explained by each components (blue bars) as well as the cumulative percentage explained (red bars). (E) Representation of all nucleosomes in two-dimensional modification space. In the left panel, each point represents a nucleosome plotted according to the relative level of the first principal component (*x*-axis) and second principal component (*y*-axis) for the modification pattern. The right panel is a three-dimensional plot showing density of points along the plane.

The analysis presented above is highly informative, but is based on aggregated data for many promoters, and thus may obscure interesting underlying phenomena. A more informative approach would be to examine the distinct modification patterns at individual nucleosomes. We defined the modification pattern of each nucleosome as the median hybridization value, for each measured antibody, of the probes associated with the nucleosome (usually between six and 15 probes; see Materials and Methods). In addition, we classified nucleosomes according to their positions relative to genome annotations (Figure 3A; see Materials and Methods). We used nine annotation categories that represent nucleosomes in promoter regions, transcribed regions, and other regions (tRNA genes and autonomously replicating sequences (ARSs). These classifications are discussed further below.

Nucleosomes were clustered by modification pattern, using a probabilistic hierarchical agglomerative clustering procedure (see Materials and Methods). As is readily apparent from this clustering (Figure 3B), histone modification patterns span the full possible range of overall modification level, from hypo-acetylated to hyper-acetylated. Nevertheless, a striking aspect of this clustering is the limited range of observed modification patterns. Visual inspection suggests that, as previously noted [18], histone modifications are not independent of each other. Indeed, the matrix of correlations between the 12 modifications shows that there are two groups of strongly correlated acetylations (Figure 3C).

To better understand the effective number of degrees of freedom among the 12 dimensions available, we performed a principal component analysis (see Materials and Methods). Principal component analysis is a technique used to transform a large number of possibly correlated variables to a smaller number of uncorrelated variables, and thereby identify the number of independent dimensions in a dataset. As suggested by the observation above, 81% of the variance in histone modification patterns is captured by the first two principal components (Figure 3D). Moreover, if we examine only the nine acetylations, we can explain 90% of the variance using two components (unpublished data). The first principal component corresponds to overall level of histone modification (Figure S5). The second principal component corresponds to the relative levels of the two groups of histone modifications—the transcription-associated modifications that occur in 5′ to 3′ gradients over coding regions, and the group of acetylations characterized by short hypo-acetyl domains surrounding TSS (Figure S5). By projecting each nucleosome to a point in the plane spanned by the first two principal components (Figure 3E), we can visualize the range of observed modifications. There is a large region of allowable modifications that is spanned continuously by different nucleosomes. These results suggest that, at the level of cell populations, there are no discrete states for nucleosome modifications. Instead, nucleosome modification patterns occur continuously over a large range of possible space, though this two-dimensional space is dramatically simplified compared to the 12 dimensions available. In other words, nucleosomes have continuous variation, both in the total level of acetylation, and in the relative ratio of the two groups of modifications, but they do not show much complexity beyond these two axes.

### Specific Chromosomal Locations Are Associated with Characteristic Histone Modifications

**Figure 4:**
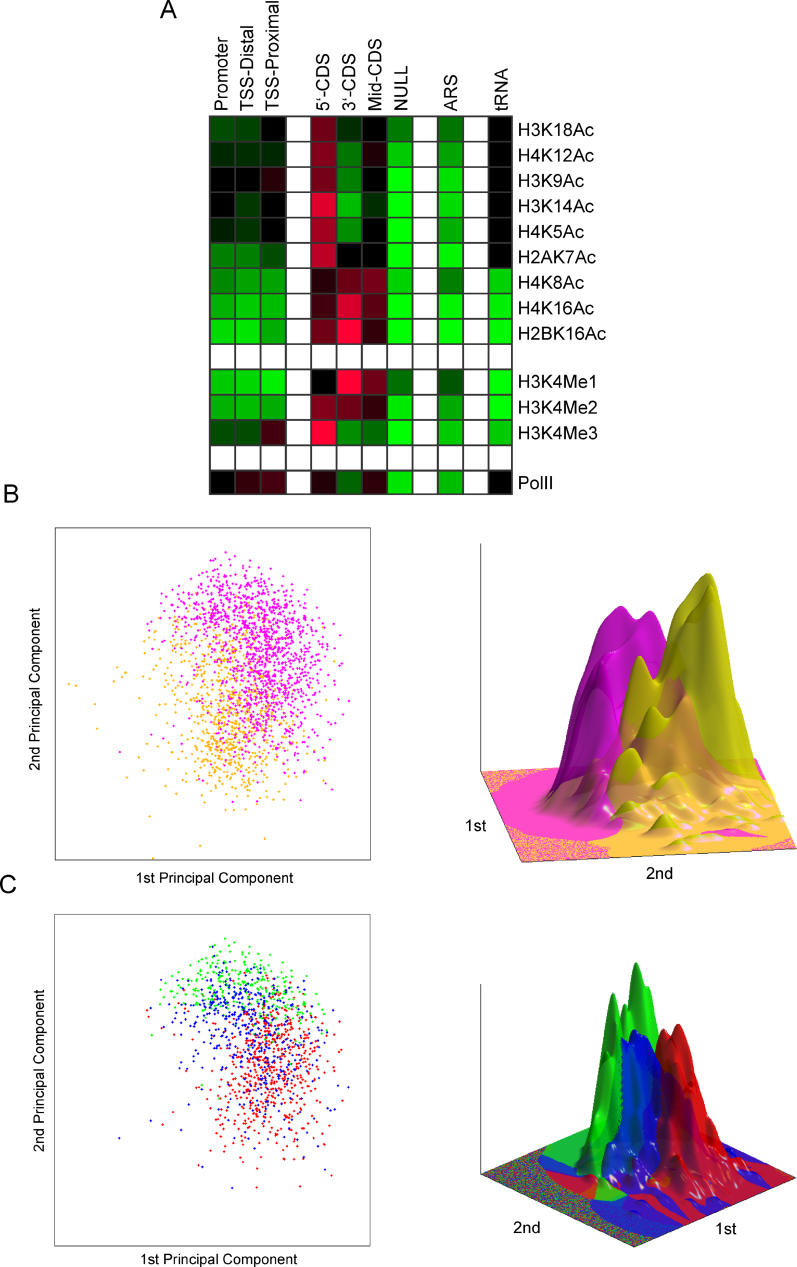
Nucleosome Modifications Relate to Nucleosome Position (A) Analysis of differential modification for each class of nucleosomes. Rows correspond to specific modifications, and columns correspond to genomic locations. Each cell is coloured by the average modification level of nucleosomes with this annotation. Non-significant (using false discovery rate of 95% on *t-*test *p*-values) cells are blackened. (B) Promoter nucleosomes (orange) significantly differ from coding region nucleosomes (pink) in their histone modifications pattern. The left panel shows the two types of nucleosomes as points in the plane, where the *x*-axis represents the level of the first principal component, and the *y*-axis represents the second principal component. The right panel shows the density within each class. (C) Distinction between nucleosomes in transcribed regions. Colours denote 5′-end (red), middle (green), and 3′- end (blue) nucleosomes. Visualization is as described in (B).

Notable in Figure 3B is an association of particular modification patterns with specific genomic locations. For example, Cluster 2 consists of hypo-acetylated nucleosomes that are predominantly located within promoter regions and at the 3′ ends of coding regions. We systematically explored these correlations by testing the modification data for statistically significant, location-specific differences in the levels of each modification type (Figure 4A). For example, promoter nucleosomes are globally hypo-acetylated in residues H2A K7 (presumably due to the enrichment of Htz1), H2B K16, and H4K8 and K16 (and, to a lesser extent, H3K18), and are depleted of mono- and di-methylated H3K4. Nucleosomes at 5′ ends of coding regions are enriched for H3K4Me3, as well as H3K18Ac, H4K12Ac, H3K9Ac, H3K14Ac, H4K5Ac, and H2AK7Ac. When we examine the modification patterns of individual nucleosomes in the two-dimensional principal component plot, we can clearly distinguish nucleosomes in promoter regions from those in transcribed regions (Figure 4B). Moreover, of the nucleosomes in transcribed regions, we can distinguish among nucleosomes in the 5′ end, the middle, and the 3′ end of the transcribed region (Figures 4C and S6).

These results show that specific genomic regions are characterized by distinct modification patterns, with little overlap in modification types between the different regions. We conclude that the histone modification patterns are highly informative about the location of nucleosomes along the chromosome, and suggest that, in yeast, nucleosome modification patterns, like nucleosome positioning, exhibit local variation around a basic stereotype that is determined by the chromosomal location.

### Variation in Modifications Occurring over Transcribed Regions is Predictive of Transcription Levels

**Figure 5:**
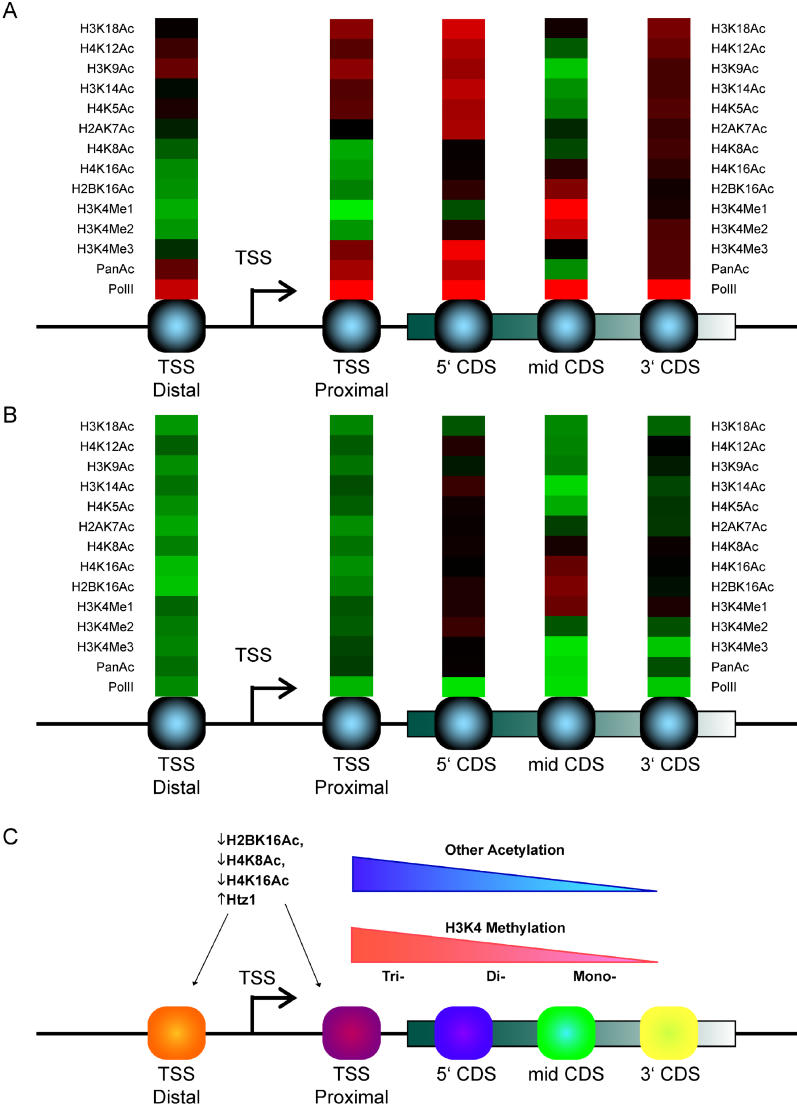
Nucleosome Modifications Partitioned by Location and by Transcription Level (A) Modification patterns of nucleosomes associated with actively transcribed genes. Genes with high levels of PolII occupancy were grouped, and the modification data for the indicated nucleosome types were averaged. (B) Modification patterns of nucleosomes associated with poorly transcribed genes, grouped as in (A), except that genes with low levels of PolII were selected. (C) Schematic view of yeast chromatin architecture. Cartoon view showing chromatin structure of an arbitrary yeast gene. Yeast genes are typically characterized by an upstream nucleosome-free region, which serves as the transcriptional start site [29]. Surrounding this nucleosome-free region are two nucleosomes that exhibit low levels of acetylation at H2BK16, H4K8, and H4K16, and that carry Htz1 in place of the canonical H2A (unpublished data). The remaining acetylations occur in a gradient from 5′ to 3′ over actively transcribed genes. Similarly, actively transcribed genes exhibit a gradient of H3K4 methylation, with trimethylation occurring at the 5′- ends of genes, and di- and mono-methylation occurring over the middle of the coding region. Nucleosomes are coloured to emphasize the different average modification patterns at each indicated location.

While nucleosomes at different locations are associated with statistically different modification patterns, the correlations are imperfect, as a given nucleosome modification pattern can clearly be found in multiple locations (Figure 4B and 4C). This imperfect association might be due to differences in expression level of the coding regions examined. We therefore separated nucleosome locations (5′ coding, etc.) into bins according to the PolII activity level of the associated transcription unit. Figure 5A shows the modification pattern of each of five nucleosomes (defined by position) for highly PolII-enriched genes, while Figure 5B shows this pattern for PolII-depleted genes. This view emphasizes both the distinction between nucleosomes at various genomic locations (as seen in aggregate in Figure 4) and the transcription-associated variation in the modification pattern at a given location. Figure 5C shows a cartoon of the chromatin structure of an arbitrary yeast gene.

**Figure 6:**
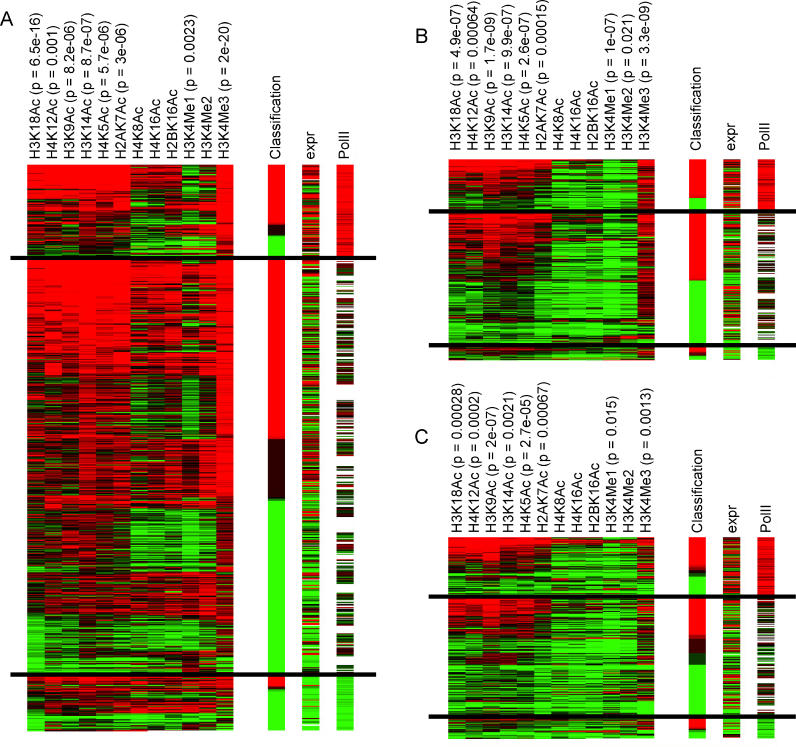
Nucleosome Modifications Relate to Transcription Level (A) Classification plot of nucleosomes in 5′-coding regions according to PolII occupancy. A classifier was trained to distinguish between nucleosomes with high and low PolII occupancy, and evaluated using leave-one-out cross-validation. Each row corresponds to one nucleosome. Nucleosomes are split into three groups associated with genes corresponding to high, intermediate, and low PolII occupancy level (from top to bottom, respectively). The left 12 columns denote modification patterns of each nucleosome. Modifications with significant differences between high and low nucleosomes are marked with the *p*-value determined by *t*-test. Colours denote relative acetylation/methylation levels. The rightmost three columns correspond to the classifier's prediction of transcription, the expression level (mRNA abundance; see Materials and Methods) and the PolII occupancy of genes. The average accuracy of random classification was 60.71%, with a standard deviation of 4.3%. Accuracy of classifier was 75.38% (*p* < 0.0001). (B) Classification plot of TSS proximal nucleosomes, labelled as in (A). The average accuracy of random classification was 62.45%, with a standard deviation of 4.75%. Accuracy of classifier was 72.8% (*p* = 0.0004). (C) Classification plot of TSS distal nucleosomes; as in (A). The average accuracy of random classification was 65.79%, with a standard deviation of 4.22%. Accuracy of classifier was 58.4% (*p* = 0.9333).

To further explore the relationship between transcription activity and modification pattern at a given location, we tested each location for modifications that were significantly associated with high or low transcription. For example, we consider the nucleosomes near the 5′ ends of those genes with extreme levels of PolII enrichment or depletion (Figure 6A). Consistent with results shown in Figures 2C and 5A and 5B, we see that levels of mono- and tri-methylation of H3K4, as well as the acetylation level of H3K9, H3K14, H2A K7, H4K5, and H4K12 have significant differences between these two classes of 5′ coding region nucleosomes (*p* < 0.01 using *t*-test). We trained a classification method that examines these modifications and predicts whether the nucleosome is part of an expressed coding region or not. We evaluated this classifier using leave-one-out cross-validation (see Materials and Methods) to estimate its accuracy on unseen examples. This evaluation shows that the classifier is correct on 75.4% of the nucleosomes in the training set (compared to 60.1% when nucleosomes labels are randomly permuted; *p* < 0.0001). Thus, although expression values are not perfectly encoded by histone modifications, they are clearly reflected in them. We see a similar pattern if we examine nucleosomes in the middle of coding regions (Figure S7). In this case the accuracy is 82.7% (compared to 61.3% by chance; *p* < 0.0001). Notably, the set of significant modifications in this case is different, and in fact two of the transcription-independent modifications, H4K8 and K16, are both slightly anticorrelated with transcription here.

These results indicate that over coding regions, variation in histone modification patterns is associated with transcription level. For example, the transcription-associated modifications are globally enriched at the 5′ ends of genes, and the level of these modifications is correlated with transcription level. To explore whether these results hold true for nucleosomes that are not found over transcribed regions, and to thereby test the idea that upstream histone modifications control gene expression, we repeated the classification analysis for nucleosomes surrounding the TSS (Figure 6B and 6C), which are modified in similar ways (Figure 4A) with the exception that the gene-proximal nucleosome is associated with DNA passaged by RNA polymerase, while the gene-distal nucleosome is not. Here, we found that the gene-proximal nucleosome indeed carries information about transcription level—a classification method tested using this nucleosome correctly identified 72.8% of gene expression patterns (as compared with 62.4% by chance; *p* = 0.0004). In contrast, the gene-distal nucleosome, which is not subjected to the passage of RNA polymerase and associated modifying enzymes, fails to accurately classify transcription levels (58.4%, as compared with 65.7% expected by chance), demonstrating that modification patterns associated with transcribed regions provide a much better predictor of transcription levels than do upstream modification patterns.

### Modifications Associated with Transcriptional Regulators

**Figure 7:**
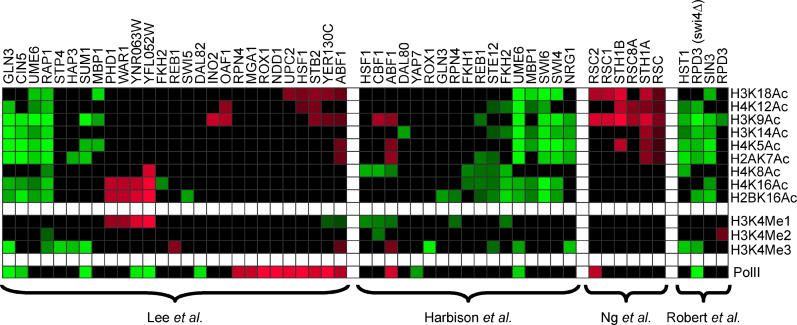
Histone Modifiers Analysis of differential modification of nucleosomes associated with various transcriptional regulators. Promoter nucleosomes located near binding sites of the indicated factors were tested for enrichment of all modifications relative to the overall promoter modification pattern. Each cell is coloured by the average modification level of nucleosomes with this annotation. Non-significant cells (using false discovery rate of 95% on *t-*test *p*-values) are blackened. Localization data are taken from the indicated studies [39–42].

The observed modifications at the two TSS nucleosomes might be either a prerequisite for PolII recruitment or a consequence of this step. Since we measure modification in a single condition, we cannot directly resolve this question. However, we can gain additional insight by examining nucleosomes in promoters reported to be bound by specific chromatin remodelers or by specific transcription factors. Using the results of several recent ChIP studies [39–41], we compiled a set of target promoters for each factor (see Materials and Methods). We then tested for distinct patterns in the promoter nucleosomes. In addition, we analyzed nucleosomes around putative transcription factor binding sites [42] (see Materials and Methods). Our results highlight specific factors that are significantly associated with specific modifications (Figure 7). For instance, we see that promoters of genes bound by the repressor Ume6 are significantly hypo-acetylated at most positions. This finding correlates with previous observations demonstrating recruitment of the HDAC Rpd3 by Ume6 [43,44]. Another interesting example is the significant hyper-acetylation of several positions among the targets of the Rsc remodeling complex. These include H3K9 and, to a lesser extent, H4K12, H3K14, and H4K5. Recently, mutants in the Rsc complex were shown to interact genetically with K14 mutations, a finding supported by binding of the complex to K14-acetylated H3-tail peptides [45].

### Modification Boundaries Occur Near Transcriptional Start Sites

**Figure 8:**
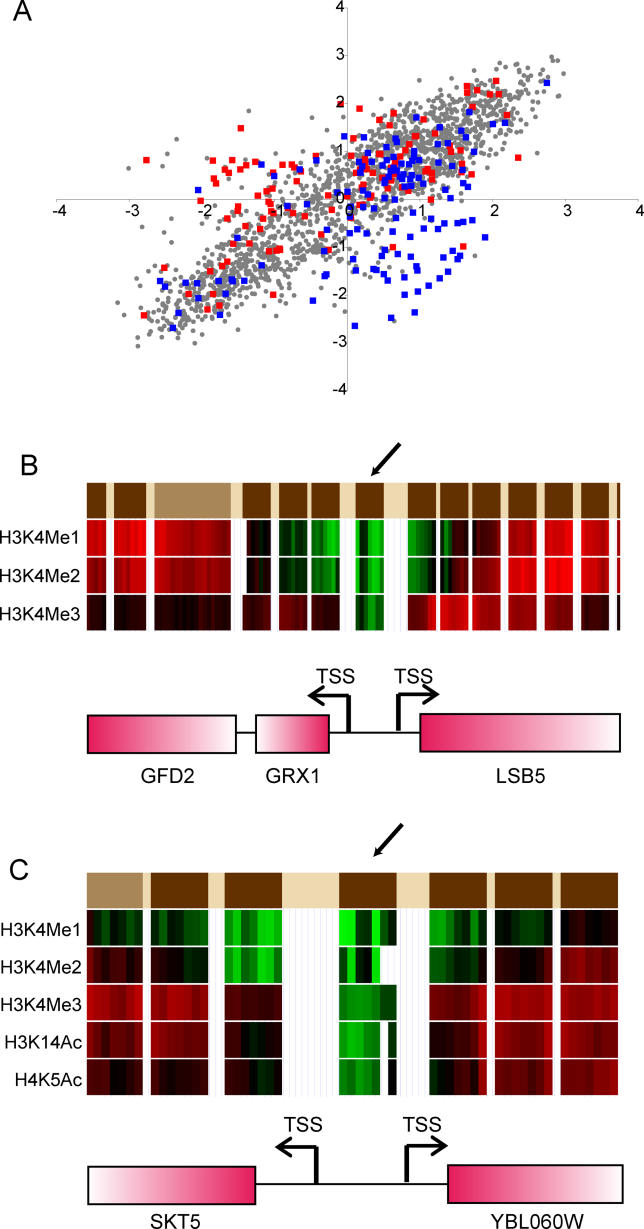
Modification Boundaries (A) H3K4Me3 boundaries occur across TSSs. The *x*-axis represents the level of H3K4Me3 for a given nucleosome, and the *y*-axis represents the level of this modification for the preceding nucleosome. Pairs of nucleosomes flanking the TSS for a gene on the W strand are plotted as blue squares, and pairs flanking TSSs for genes on the C strand are plotted as red squares. Remaining nucleosome pairs are plotted as grey circles. (B) Example of a punctate nucleosome. Histone modification plotted as in Figure 1B for a subset of histone modifications. Arrow indicates a nucleosome whose modification pattern differs significantly for H3K4Me3 from nucleosomes to either side. Gene names are as labelled. (C) Example of a punctate nucleosome, labelled as in (B).

The availability of histone modification data at single nucleosome resolution allows analysis of the extent to which modification patterns occur discretely or in broad domains. As noted above and previously reported [44], histones can be deacetylated in a localized manner. However, visual inspection reveals that at locations farther away from the TSS, most histone modifications occur in broad domains. To further investigate this, we searched for sharp boundaries to histone modification domains by identifying pairs of nucleosomes between which a dramatic change occurs (increase or decrease of two standard deviations at one of the tail positions). We found ~100 boundaries for each modification (from 82 to 108). We then examined the locations of these boundaries, finding that most were located adjacent to TSSs. For example, boundaries for modifications associated with transcription, such as H3K4 tri-methyl, occurred across the TSS. This is visualized in Figure 8A, a scatterplot of K4 tri-methylation for adjacent nucleosomes (*x*-axis shows tri-methylation for nucleosome N, *y*-axis shows tri-methylation of N-1). The majority of nucleosomes show high correlation for this modification between adjacent nucleosomes, though there are two small groups of anticorrelated nucleosomes, indicating methylation boundaries. Pairs of nucleosomes that fall to either side of the TSS were plotted separately (grouped according to which strand the gene falls on), showing that most of the K4 tri-methyl boundaries occur at the TSSs, as expected.

We also examined “punctate” nucleosomes—those differing significantly in modification type from the two nucleosomes to either side. We found 44 nucleosomes with a punctate pattern of at least one of the 12 modifications in this study. Examples of punctate nucleosome are shown in Figure 8B and 8C. Most nucleosomes that exhibit this characteristic are found upstream of the TSS. In many cases, this is clearly due to the location of the nucleosome between two TSSs, leading to a single nucleosome exhibiting no transcription-associated modifications, surrounded by nucleosomes with the characteristic transcriptional modifications.

## Discussion

### Profiling Histone Modification at the Mononucleosome Level

We have mapped, at single-nucleosome resolution, 12 histone modifications in actively dividing cultures of *S. cerevisiae*. This, along with the translational positioning of nucleosomes described previously [29] and location studies on the H2A isoform Htz1 (unpublished data), provides a draft sequence (see below) of the primary structure of half a megabase of yeast chromatin. We wish to stress the importance of the high resolution of our method for deconvoluting the results of previous studies on histone modification. The use of ~1-kb intergenic and coding probes in standard microarray studies reports on mixtures of multiple nucleosomes. For example, we show that the two nucleosomes immediately adjacent to the TSS are generally deacetylated at H4K16, whereas surrounding nucleosomes are often highly acetylated (Figure 2B). As a result, the acetylation level measured in standard microarray studies will depend on the length of the 5′ untranslated region (which is especially confounding, as this correlates with functional classifications of the encoded genes [46]); the length of the entire intergenic region probed; and the nature of the intergenic region (divergent or parallel genes), as the deacetyl signals from the TSS will be diluted by these additional nucleosomes in a complicated way. Furthermore, the ~300–500-bp standard shear size used in microarray studies results in some sampling of additional nearby nucleosomes outside the borders of the microarray spot. Our methodology eliminates all these confounding variables and also controls for local variation in nucleosome density, thus dramatically simplifying modification mapping.

We note, however, that our study is subject to the same issues with antibody specificity that remain a crucial limitation of ChIP studies—the epitope accuracy of any ChIP study is determined by the specificity of the antibodies used. We used the state-of-the-art in antibodies (see Materials and Methods), but improvements in antibody specificity may improve the fidelity of these experiments. In addition, ensemble measurements such as those presented here necessarily provide population averages, and we cannot rule out the possibility that small subpopulations of cells in different phases of the cell cycle, or in different epigenetic states, might be characterized by modification patterns that are obscured in the population average. Finally, this study does not provide a complete sequence of chromatin's primary structure in our tiled region. A complete view of the primary structure requires the addition of all additional modifications, including core domain modifications, and, ideally, the conformations of the nucleosomes studied.

### Histone Tail Modifications Occur in Two Groups that Vary Quantitatively

This mapping has allowed us to investigate combinatorial questions raised by the framing of histone modifications as a “code.” Most importantly, we have shown that many histone modifications are highly correlated with one another, resulting in few discrete histone modification patterns. However, we cannot say whether these modifications occur in the same nucleosome or whether the correlations are due to a mixture of partially modified nucleosomes at a given location. Some modified residues may be correlated because histone-modifying enzymes are not strongly residue-specific [8,47], whereas other correlations may be due to histone-modifying enzymes that are either recruited to chromatin by association with other types of modification, or preferentially act on tails carrying another modification [48–50]. Still other modifications may be correlated because the relevant modifying enzymes may be targeted by association with similar complexes, such as RNA polymerase [23,51]. These correlations suggest a high level of redundancy in yeast histone modification, implying that the code is extremely simple, carrying only a tiny fraction of the maximum possible amount of information. Indeed, as principal component analysis shows, we can compress the 12-dimensional space of possible modification patterns onto two main axes, with only a minor loss of accuracy.

This raises the important question of why so many different modifications occur in the cell, yet such a small subset of combinations is used. We suggest only a few possible answers. First, the loss of a positive charge that occurs with lysine acetylation should reduce the free energy of interaction with a negative charge by approximately 1–3 kcal/mol. Thus, loss of multiple positive charges could lead to much greater free energy changes in an interaction, and to a much more pronounced change in interactions than would be caused by a single acetylation. Furthermore, we note that at any given nucleosome location the quantitative level of acetylation varies, allowing for the possibility of “rheostat”-like control of transcription levels. This is consistent with recent mutagenesis studies showing that transcriptional response to H4K→R mutations is largely continuous and analogue, rather than discrete and digital [24]. Second, it is possible that multiple modifications occur together in order to cause several distinct required events to occur, whether they be co-occurring structural changes in the nucleosome or the 30-nm fibre, or recruitment of protein complexes that function together. This has been observed at the human interferon-β promoter, wherein activation of the promoter causes Gcn5-dependent acetylation of H3K9/14 and H4K8, whose acetylation recruits TFIID and hSWI/SNF, respectively [52]. If these protein complexes tend to function together, then the recruiting modifications will be correlated. Third, if modifications that occur together at steady-state do not occur simultaneously, but rather in a temporal cascade [6], this enables the possibility of complex signal filtering behaviour. For example, if one histone acetylase were to acetylate a single lysine, and that acetyl-lysine were to recruit a distinct histone acetylase that acetylated another lysine, then a requirement for both acetylations for transcription to occur would produce a low-pass filter. This filter would reject transient spikes in signalling pathways and allow transcriptional outcomes only in response to sustained signalling. A careful examination of the temporal response of histone modifications to signalling will help determine if this might occur for the correlated modifications. Finally, if one modification recruits enzymes that modify the remaining residues, then having multiple modifications allows for switch-like behaviour [53,54].

### Stereotyped Promoter Architecture

One of the two groups of histone modifications exhibits a striking, stereotyped pattern in promoter regions. Nucleosomes immediately adjacent to the TSS are hypo-acetylated at H2BK16, H4K8, and H4K16. This hypo-acetylation does not correlate with transcription levels, and the inability of the histone modification pattern at the gene-distal TSS-adjacent nucleosome to accurately reflect transcriptional activity of the associated gene (Figure 6C) does not support the idea that upstream modifications are causal for transcription.

In separate work, we have identified this di-nucleosomal domain that flanks the TSS as highly enriched for the H2A isoform Htz1 (demonstrating that these nucleosomes do not appear deacetylated due to some artifactual difficulty with immunoprecipitation). Also, this enrichment is independent of transcription (unpublished data). In other words, the majority of promoter nucleosome-free regions in yeast are surrounded on either side by nucleosomes with hypo-acetylated H2BK16, hypo-acetylated H4K8 and K16, and Htz1 in place of H2A. These results raise two questions: how does this domain arise, and what is its functional role in transcription?

Previous reports have shown that Rpd3 deacetylates one to three nucleosomes when recruited to promoters [44], consistent with the width of this deacetylation domain. However, the generality of the pattern observed here suggests that multiple distinct deacetylases function in this localized manner, because Rpd3 is present at only a subset of the promoters analyzed [31,43]. Alternatively, it is possible that these nucleosomes turn over rapidly (due to the presence of some assembly of chromatin-remodelling activities at promoters), and that the histone isoform and modification pattern exhibited reflects the composition of free histones in the nucleoplasm. In either case, the function of this domain remains elusive at present.

### Relationship of Histone Modifications to Transcription

We have described a group of histone modifications that co-occur, and that are preferentially found at the 5′ ends of actively transcribed genes. This relationship between histone modification patterns, location relative to coding regions, and transcript abundance, would be expected if histone modification played a largely passive, rather than instructive, role in transcription, with nucleosomes being modified by various enzymes associated with RNA polymerase. This is clearly the case, for example, for PolII-associated Set1, which is responsible for the correlation between H3K4 tri-methylation over the 5′ end of coding regions and corresponding transcription levels. A similar type of mechanism appears to hold for the Set2-mediated tri-methylation of H3K36, which occurs over transcribed genes [55]. However, mutant studies have shown abundant transcriptional defects associated with mutations in histone-modifying enzymes [56,57]. These studies cannot determine whether histone modification is instructive or permissive for transcription—in other words, whether histone modifications initiate a chain of events that result in transcription, or whether that gene is associated with a non-permissive chromatin structure that must be antagonized using the modification in question. We suggest that the transcription-associated modifications play a permissive role in gene expression, and that the transcriptional defects in histone-modification mutants result from a partial inability of RNA polymerase to transit unmodified nucleosomes [58,59], or to a failure to recruit factors required for efficient transcription [60]. However, we do not rule out the possibility that histone modifications play both roles, with an initial mark that is causal for a transcription pattern subsequently “erased” by modifications occurring with the resultant transcription.

### The Histone Code

Taken together, these results do not support a model for the histone code in which a vast set of widely varying modification combinations play complicated instructive roles in transcriptional regulation. Instead, these results further extend genome-wide studies in *Drosophila,* which show that histone modifications occur in few independent combinations [25], and suggest that these patterns are often the result, rather than the cause, of transcription. These results therefore emphasize a role for modifications of the histone tails as facilitators of transcription. It will be of great interest in future studies to assay the dynamic nature of histone modifications during changes in transcription, and the establishment of histone modification patterns during DNA replication.

## Materials and Methods

### Yeast culture

An aliquot of 450 ml of BY4741 *bar1*Δ cells was grown to an A_600_ OD of 0.9 in 2-L flasks shaking at 200 rpm in a 28 °C water bath. Formaldehyde (37%) was added to a 1% final concentration, and the cells were incubated for 15 min at 25 °C, shaking, at 90 rpm. Then, 2.5 M glycine was added to a final concentration of 125 mM, to quench the formaldehyde. The cells were inverted and let to stand at 25 °C for 5 min. The cells were spun down at 3,000 × g for 5 min at 4 °C and washed twice, each time with an equal volume of ice-cold sterile water.

### Micrococcal nuclease digestion

The cell pellets were resuspended in 39 ml Buffer Z (1 M sorbitol, 50 mM Tris-Cl [pH 7.4]), 28 μl of β-ME (14.3 M, final concentration 10 mM) was added, and cells were vortexed to resuspend. Then, 1 ml of zymolyase solution (10 mg/ml in Buffer Z; Seikagaku America, Falmouth, Massachusetts, United States) was added, and the cells were incubated at 28 °C, shaking at 200 rpm, in 50-ml conical tubes, to digest cell walls. Spheroplasts were then spun at 3,000 × g, 10 min, at 4 °C. Spheroplast pellets were resuspended and split into aliquots of 600 μl of NP-S buffer (0.5 mM spermidine, 1 mM β-ME, 0.075% NP-40, 50 mM NaCl, 10 mM Tris [pH 7.4], 5 mM MgCl_2_, 1 mM CaCl_2_) per 90-ml cell culture equivalent. Forty units of micrococcal nuclease (Worthington Biochemical, Lakewood, New Jersey, United States) were added, and the spheroplasts were incubated at 37 °C for 20 min—this was determined in initial titrations to yield > 80% mononucleosomal DNA (see Figure S1), but to repeat these results an independent titration should be carried out as a preliminary study. The digestion was halted by shifting the reactions to 4 °C and adding 0.5 M EDTA to a final concentration of 10 mM.

### ChIP

All steps were done at 4 °C unless otherwise indicated. For each aliquot, Buffer L (50 mM Hepes-KOH [pH 7.5], 140 mM NaCl, 1 mM EDTA, 1% Triton X-100, 0.1% sodium deoxycholate) components were added from concentrated stocks (10–20×) for a total volume of 800 μl per aliquot. Each aliquot was incubated with 80–100 μl of 50% Sepharose Protein A Fast-Flow bead slurry (Sigma, St. Louis, Missouri, United States) equilibrated in Buffer L for 1 h on a tube rotisserie rotator. The beads were pelleted with a 1-min spin at 3,000 × g, and approximately 2.5%–5% of the supernatant was set aside as ChIP input material. With the remainder, antibodies were added to each aliquot (20% of a 450-ml cell culture) in the following volumes: 25 μl anti-H3K4Me1 Ab (affinity purified; Abcam, Cambridge, Massachusetts, United States), 6 μl anti-H3K4Me2 Ab (affinity purified; Abcam), 6 μl anti-H3K4Me3 Ab (affinity purified; Abcam), 4 μl anti-H4K16Ac Ab (whole antiserum; Abcam), 9 μl anti-H4K5Ac Ab (whole antiserum; Abcam), 3 μl anti-H3K14Ac Ab (whole antiserum; Upstate Cell Signaling Solutions, Charlottesville, Virginia, United States), 3 μl anti-H2AK7Ac Ab (whole antiserum; Upstate), 2 μl, anti-H4K8Ac Ab (whole antiserum; Abcam), 15 μl, anti-H4K12Ac Ab (whole antiserum; Abcam), 25 μl anti-Ac Ab (whole antiserum; Abcam), 16 μl anti-H3K9Ac Ab (affinity purified; Abcam), 25 μl anti-H2BK16Ac (L) (whole antiserum; Abcam), and 3 μl anti-H3K18Ac Ab (whole antiserum; gift of M. Grunstein). We also used 3 μl of a distinct antibody to H4K16Ac (whole antiserum; gift of M. Grunstein) to assess specificity of different sources of antibody. Replicates using this antibody were as correlated with each other as they were with replicates using the Abcam antibody.

These were incubated, rotating, overnight (∼16 h), after which the sample was transferred to a tube containing 80–100 μl of 50% Protein A bead slurry. The sample was incubated with the beads for 1 h for the immunoprecipitation, after which the beads were pelleted by a 1-min spin at 3,000 × g. After removal of the supernatant, the beads were washed with a series of buffers in the following manner: 1 ml of the buffer would be added, and the sample rotated on the tube rotisserie for 5 min, after which the beads would be pelleted in a 30-s spin at 3,000 × g and the supernatant removed. The washes were performed twice for each buffer in the following order: Buffer L, Buffer W1 (Buffer L with 500 mM NaCl), Buffer W2 (10 mM Tris-HCl [pH 8.0], 250 mM LiCl, 0.5% NP-40, 0.5% sodium deoxycholate, 1mM EDTA), and 1× TE (10 mM Tris, 1 mM EDTA [pH 8.0]). After the last wash, 125 μl of elution buffer (TE [pH 8.0] with 1% SDS, 150 mM NaCl, and 5 mM dithiothreitol) was added to each sample, and the beads were incubated at 65 °C for 10 min, with frequent mixing. The beads were spun for 2 min at 10,000 × g, and the supernatant was removed and retained. The elution process was repeated once for a total volume of 250 μl of eluate. For the ChIP input material set aside, elution buffer was added for a total volume of 250 μl. After overlaying the samples with mineral oil, the samples were incubated overnight at 65 °C to reverse cross-links.

### Antibody specificity

A significant concern with ChIP studies is the epitope specificity of the antibodies used. High correlations between different modifications could arise if two antibodies cross-reacted. We note four reasons that this is unlikely to be a major problem for this study. First, if antibodies did indeed cross-react, then the resulting profiles should look like some weighted average (depending on relative affinities of the two antibodies) of the two “pure” profiles. If there were a third modification pattern (besides what we term the *transcription-dependent* and *transcription-independent* patterns), then the two antibodies in question would be expected to show a third *mixed* pattern, distinct from the two patterns described, and this was not observed. On the other hand, if only two true patterns do exist but there is cross-reactivity for antibodies, the mixed profile is expected to show a 5′ gradient of acetylation, along with two deacetyl nucleosomes adjacent to the TSS. This pattern was seen for H2AK7, but, as we note, this is likely due to the replacement of H2A with Htz1 at the TSS-adjacent nucleosomes. Furthermore, this pattern was not seen for the H3K14 antibody, which recognizes lysine in the context of a similar site to that of H2AK7 (GGKA). So we do not believe that these antibodies are cross-reacting.

Second, we repeated experiments for one of the epitopes in this study (H4K16) with two distinct antibodies, and the results were indistinguishable. One of these antibodies, from the Grunstein lab, was previously tested for cross-reactivity by attempting ChIP from strains carrying the H4K16R mutation [37].

Third, there are two pairs of antibodies for which cross-reaction is most likely to be a concern: H4K5 and K12 (both lysines occur in the context of GKGG), and H2AK7 and H3K14 (both occur in the context of GGKA). However, within each pair, the two antibodies are more highly correlated with other antibodies in their group than with the other antibody with a similar recognition site (see Figure 3C). If these antibodies had cross-reacted, then their profiles should be the most highly correlated. In addition, technical literature from Upstate shows that both the H2AK7 and H3K14 acetylation antibodies fail to immunoprecipitate DNA from yeast strains carrying the appropriately mutated recognition site.

Finally, it is worth noting that even if a pair or two of antibodies cross-reacted, the point that histone modifications occur at reduced dimensionality would still hold. Instead of 12 dimensions reducing to two dimensions, we would say, for example, that 10 dimensions reduce to two. This is not, to our thinking, a significant change in the central message of this study. In addition, it would not challenge the other main points of the manuscript, that the two TSS-adjacent nucleosomes exhibit a stereotyped modification pattern and that most of the histone modification that correlates with transcription levels occurs over coding regions.

### Protein degradation and DNA purification

After cooling the samples down to room temperature, each sample was incubated with an equal volume of proteinase K solution (1× TE with 0.4 mg/ml glycogen, and 1 mg/ml proteinase K) at 37 °C for 2 h. Each sample was then extracted twice with an equal volume of phenol and once with an equal volume of 25:1 chloroform:isoamyl alcohol. Phase-lock gel tubes were used to separate the phases (light gel for phenol, heavy gel for chloroform:isoamyl alcohol). Afterwards, 0.1 volume 3.0 M sodium acetate [pH 5.3] and 2.5 volumes of 100% ice-cold ethanol were added, and the DNA was allowed to precipitate overnight at −20 °C. The DNA was pelleted by centrifugation at 14,000 × g for 15 min at 4 °C, washed once with cold 70% ethanol, and spun at 14,000 × g for 5 min at 4 °C. After removing the supernatant, the pellets were allowed to dry and then were resuspended in 20 μl 10 mM Tris-Cl, 1 mM EDTA [pH 8.0], and 0.5 μg of RNase A was added. The samples were incubated at 37 °C for 1 h, and then treated with 7.5 units of calf intestinal alkaline phosphatase in a 30-μl volume supplemented with NEB Buffer 3 (10× concentration of 100 mM NaCl, 50 mM Tris-HCl [pH 7.9], 10 mM MgCl_2_, 1 mM dithiothreitol). The samples were then incubated for a further 1 h at 37 °C and then cleaned up with the Qiagen MinElute Reaction Cleanup Kit (Qiagen, Valencia, California, United States), following manufacturer's directions, except with an elution volume of 20 μl.

### Linear amplification of DNA

The samples were amplified, with a starting amount of 125 ng for ChIP input materials and up to 75 ng for ChIP samples, using the DNA linear amplification method described in BMC Genomics 4:19 [32].

### Microarray hybridization

RNA produced from the linear amplification (3 μg) was used to label probe via the amino-allyl method as described at http://www.microarrays.org. Labelled probes were hybridized onto a yeast tiled oligonucleotide microarray [29] at 65 °C for 16 h, and washed as described at http://www.microarrays.org. The arrays were scanned at 5-μm resolution with an Axon Laboratories (Sunnyvale, California, United States) GenePix 4000B scanner running GenePix 5.1.

### Image analysis and data processing

Array features were filtered using the autoflagging feature of GenePix 5.1 with the following criteria defining features to be discarded: [Flags] = [Bad] Or[Flags] = [Absent] Or[Flags] = [Not Found] OrLCase([ID]) = “empty” OrLCase([ID]) = “blank” Or([SNR 635] < 3 And [SNR 532] < 3) Or[F Pixels] < 100 Or([F Pixels] < 150 And [Circularity] < 75).

The remaining features for each array were then block-normalized by calculating the average net signal intensity for each channel in a given block, and then taking the product of this average and the net signal intensity for each filtered array feature in the block. Afterwards, all block-normalized array features were normalized using a global average net signal intensity as the normalization factor.

Each histone tail modification epitope was chromatin-immunoprecipitated in three to six biological replicates, with additional technical replicates of the microarray hybridizations. Outlying replicates were removed (with a minimum remainder of three replicates), and the median was calculated and used for subsequent data analysis.

### Normalization of modification and PolII data

Each assay was repeated three to six times, and median values per probe were calculated. Measurements for each antibody were first log (base 2) transformed and then normalized (to mean of zero and variance of one).

### Data availability

Data can be viewed at http://compbio.cs.huji.ac.il/Nucs. Data are downloadable at http://www.cgr.harvard.edu/chromatin, and have been deposited in GEO.

### Clustering of aligned genes

The genes were clustered using PCluster, a probabilistic hierarchical clustering algorithm [61]. Probes at locations relative to gene reference point, either beginning of coding sequence (CDS) (Figure 2A) or TSS (Figure 2B), are used as attributes of the gene. Linker probes (based on the nucleosome locations of [29]) were discarded and treated as missing values.

### Splitting genes into transcriptional groups

Each gene was assigned a transcription activity value based on the average enrichment of PolII along CDS probes. Genes with less than five CDS probes were removed to reduce noise. We then used thresholds of 0.75 and −0.75 to classify genes as highly, mid-, and untranscribed. This resulted in 75 highly transcribed genes, 192 intermediate genes, and 57 poorly transcribed genes. We also repeated the analysis presented in Figure 2C using mRNA abundance rather than PolII occupancy to bin genes (Figure S4), and the results were qualitatively indistinguishable.

### Averaging probes into nucleosomal-based data

A total of 24,947 probes were assigned to 2,288 nucleosomes using a four-probe minimum size cutoff [29]. We used the hand-called set of nucleosome positions (these were generated by inspection and adjustment of the automated hidden Markov model calls; these positions are provided in the dataset associated with [29]), as that set covered a slightly greater fraction of the genome. Results are qualitatively unchanged when only HMM calls are used (unpublished data). For each antibody, the nucleosomal values were set by the median levels of relevant probes.

### Genomic classification of nucleosomes

Nucleosomes were annotated based on their relative position to nearby genes. Nucleosomes in the first (or last) 500 bp of annotated genes were annotated as 5′ CDS (or 3′ CDS) nucleosome. Other CDS nucleosomes were annotated as mid-CDS. The two TSS adjacent nucleosomes were annotated as TSS distal (5′) and proximal (3′) nucleosomes. Nucleosomes upstream (up to 1 kb or closer to non-dubious CDSs) were annotated as promoter nucleosomes. Nucleosomes around tRNA genes (200 bp from each side) or ARS elements (200 bp from each side) were annotated as tRNA or ARS nucleosomes. Other nucleosomes were annotated as null. In certain cases, we allowed more than one annotation per nucleosome; for instance, a nucleosome between two divergent genes can be annotated as TSS-proximal for one gene, and a promoter nucleosome for another one.

### Single nucleosome clustering

Nucleosomes were clustered using PCluster [61], treating each nucleosome as a vector of 12 values.

### Principal component analysis

Principal component analysis was applied to the nucleosomal modification data of 2,288 nucleosomes versus 12 modifications using MATLAB 6.5 (rel 13) procedure “princomp.” Density visualization was done using Parzen windows density estimator with Gaussian kernels (with standard deviation of 0.3) .

### Genomic enrichment of modifications

We compared the modifications of nucleosomes affiliated with each genomic location (promoter, TSS distal, etc.) to all other nucleosomes, using a standard two-tail *t*-test. To correct for multiple hypotheses, we used a 5% false discovery rate procedure [62]. The average change was then calculated for < modification, genomic location > pairs with significant *p*-values.

### Transcription-specific modifications

To identify specific modifications at genomic locations with significant correlations to expression levels of nearby genes, we trained a classification method to predict whether a nucleosome was associated with genes enriched or depleted for PolII. To prevent biased results, we applied a leave-one-out cross-validation procedure in which the tested nucleosome was removed from the training set, and a classifier was trained on the rest of the nucleosomes and used to predict the held-out nucleosome label. We used a Naive Bayes classifier [63] using the implementation described [64]. We then classified the held-out nucleosome, based on the probability of its modification pattern under each of the classes. We computed the overall accuracy of classification and a *p*-value by repeating the same leave-one-out procedure with randomly reshuffled nucleosome labels.

### Functional classification of nucleosomes

We used recent genomic studies [39–41] and compiled a set of target promoters for each factor. We then tested the promoter and TSS-distal and TSS-proximal nucleosomes of these genes for enrichment of specific modifications. In addition, we created a subset of the target nucleosomes of Harbison et al., by restricting the nucleosomes to those up to 100 bp away from putative binding sites bound in rich growth conditions [42]. As described earlier, we compared the “bound” nucleosomes to all other promoter/TSS nucleosomes, and used a false discovery rate-corrected two-tail *t*-test.

## Supporting Information

Dataset S1 Complete Dataset Individual worksheets contain data for all individual replicates before range normalization, for combined median data organized by epitope, and for combined median data after range normalization.
(48 MB XLS).

Dataset S2 Replicate Reproducibility Data contain correlations between individual experiments for each antibody.
(24 KB XLS).

Figure S1 Digestion of Chromatin to Mononucleosomes before Immunoprecipitation Gels show micrococcal nuclease-digested DNA from multiple independent cultures used for the immunoprecipitations reported here. Molecular markers are as indicated. Blue dots indicate nucleosomal DNA used for immunoprecipitations, while green dots show sonicated DNA from the same culture. Digested DNA used for immunoprecipitation was typically > 80% mononucleosome.
(674 KB PDF).

Figure S2 Low Levels of Histone Modification over Heterochromatin Data are plotted as in Figure 1B. Chromosome III coordinates are shown above the modification data. Three panels show data for a portion of (from left to right) TelIIIL, HML, and TelIIIR. Only partial regions of the three are shown, as the remainder was not tiled due to cross-hybridization concerns [29].
(551 KB PDF).

Figure S3 Broad Patterns of Histone Modifications Data are aligned by the TSS, and plotted as in Figure 2B for all remaining modifications, as indicated.
(1.8 MB PDF).

Figure S4 Relationship of Histone Modifications to mRNA Abundance Genes were grouped into low, medium, and high mRNA abundance classes using data from competitive hybridizations of mRNA versus genomic DNA on cDNA microarrays (CLL and SLS, unpublished data). Low-abundance mRNAs were defined as those with log(2) ratios less than −1, while high-abundance mRNAs were defined as those exhibiting log(2) ratios greater than 1. Histone modification data are averaged and displayed as in Figure 2C, and results are qualitatively indistinguishable from those generated using PolII occupancy to classify genes.
(676 KB PDF).

Figure S5 Representation of the First Two Principal Components The first component (left panel) consists of all positive coefficients (plotted on the *y*-axis), and therefore captures the global magnitude of modification (both acetylation and methylation). The second component differentiates between the two groups of correlated modifications (see Figure 3C). Bars indicate different epitopes as indicated.
(512 KB PDF).

Figure S6 Principal Component Analysis of Nucleosome Modifications Data plotted as in Figure 4B and 4C, right panels.
(580 KB PDF).

Figure S7 Nucleosome Modifications Relate to Transcription Level Classification plot as described in Figure 5, using mid-CDS nucleosomes. The average accuracy of random classification was 61.27%, with a standard deviation of 5.76%. Accuracy of classifier was 82.65% (*p* < 0.0001).
(397 KB PDF).

### Accession Numbers

The Gene Expression Omnibus (GEO) (http://www.ncbi.nlm.nih.gov/geo) accession numbers for the experiments described here are GSM64526–GSM64587, GSM64591, and GSM64592, and are part of series accession number GSE2954.

## Abbreviations

ARS: autonomously replicating sequence
bp: base pairs
CDS: coding sequences
ChIP: chromatin immunoprecipitation
TSS: transcription start site
