# Supplementary material for: Single-Nucleosome Mapping of Histone Modifications in S. cerevisiae
Source: PLoS Biol. 2005 Aug 30;3(10):e328. doi: 10.1371/journal.pbio.0030328 (PMC1195719; doi:10.1371/journal.pbio.0030328)
Supplement: Supplementary file 1 [file pbio.0030328.sg001.pdf]

Rando Fig S1

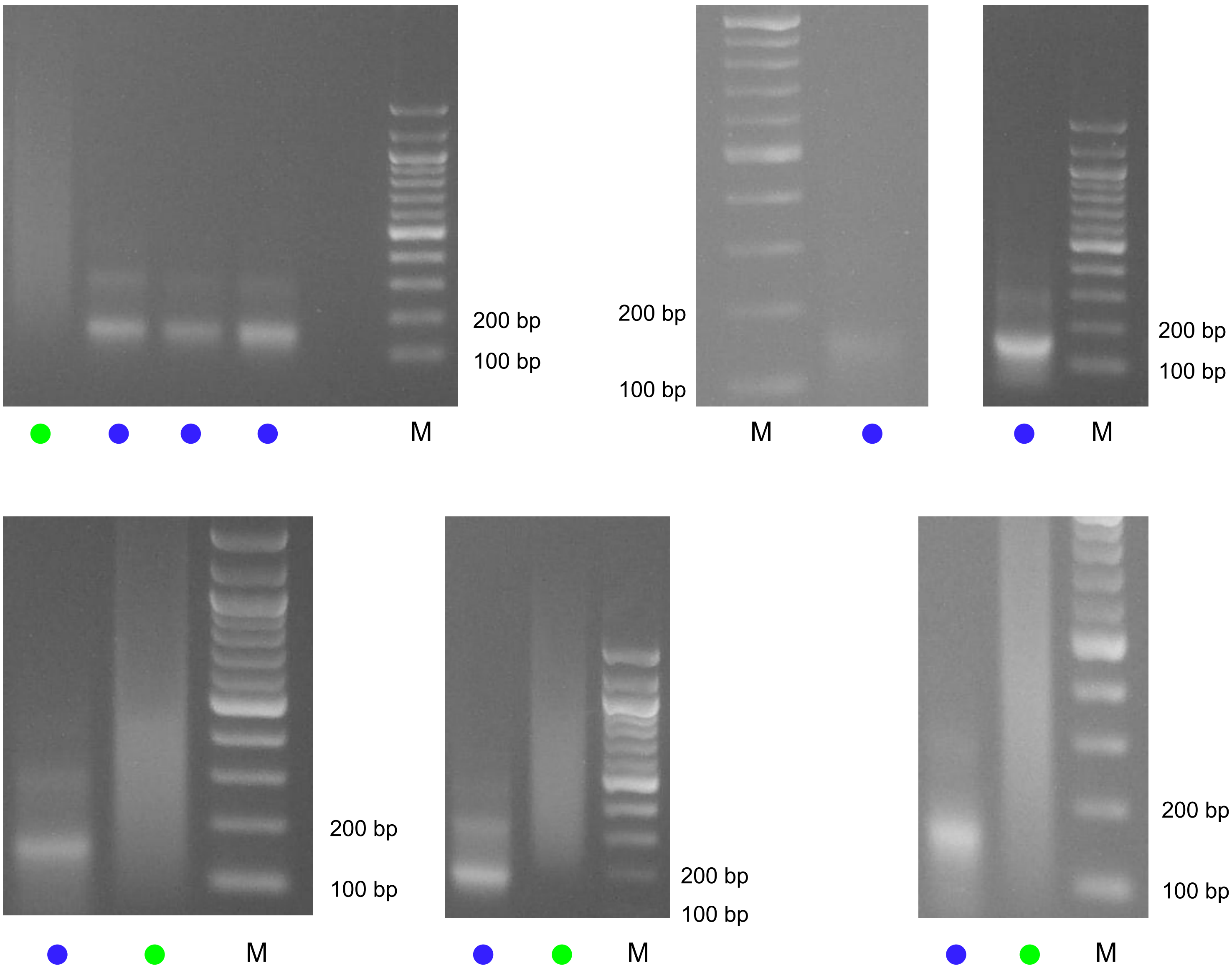
200 bp
100 bp
M
200 bp
100 bp
M
200 bp
100 bp
M
200 bp
100 bp
M
200 bp
100 bp
M
200 bp
100 bp
M
