# Supplementary material for: Single-Nucleosome Mapping of Histone Modifications in S. cerevisiae
Source: PLoS Biol. 2005 Aug 30;3(10):e328. doi: 10.1371/journal.pbio.0030328 (PMC1195719; doi:10.1371/journal.pbio.0030328)
Supplement: Supplementary file 2 [file pbio.0030328.sg002.pdf]

Rando Fig S2

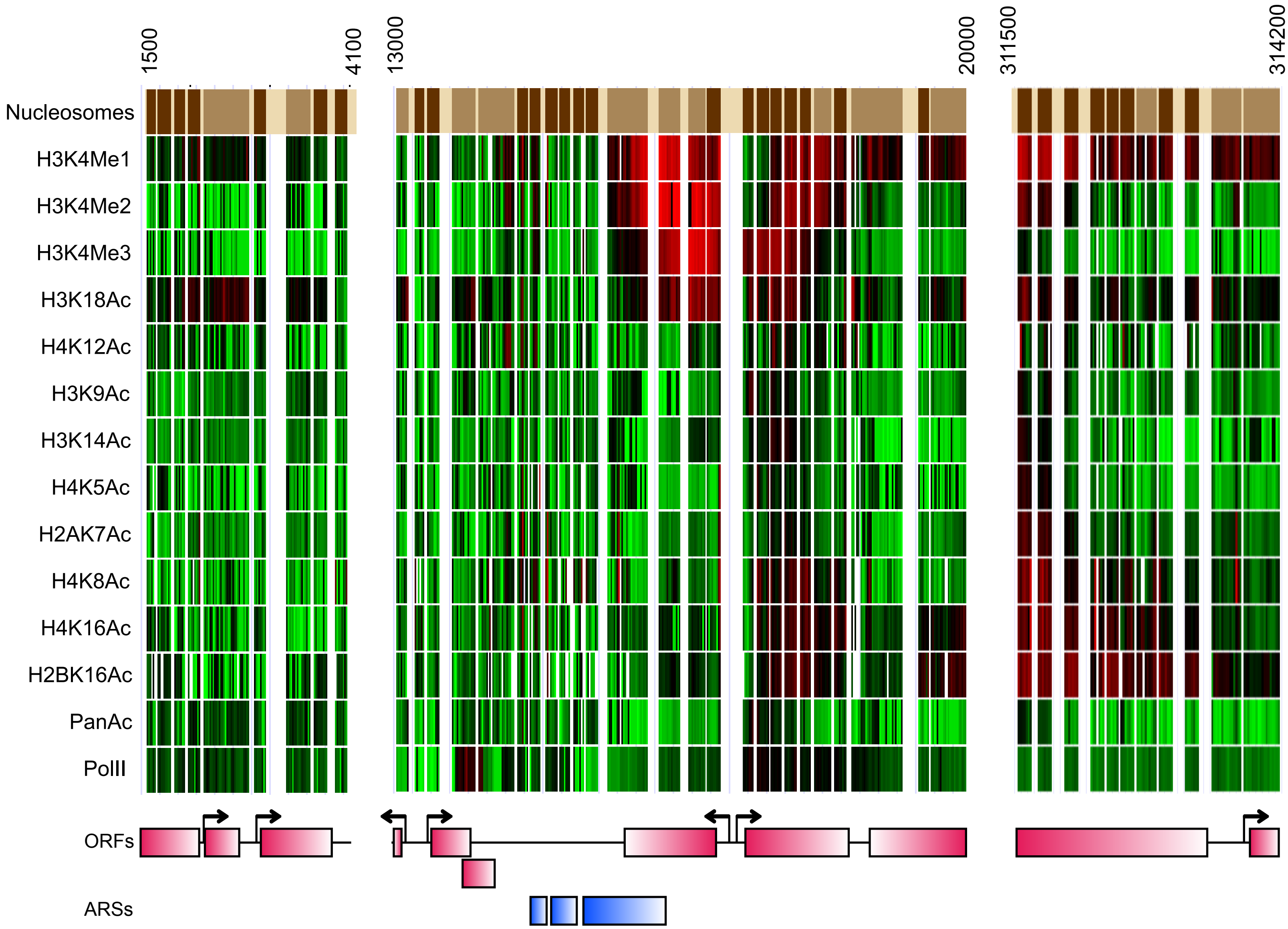
1500
4100
13000
20000
311500
314200
Nucleosomes
H3K4Me1
H3K4Me2
H3K4Me3
H3K18Ac
H4K12Ac
H3K9Ac
H3K14Ac
H4K5Ac
H2AK7Ac
H4K8Ac
H4K16Ac
H2BK16Ac
PanAc
PolII
ORFs
ARSs
