# Supplementary material for: Single-Nucleosome Mapping of Histone Modifications in S. cerevisiae
Source: PLoS Biol. 2005 Aug 30;3(10):e328. doi: 10.1371/journal.pbio.0030328 (PMC1195719; doi:10.1371/journal.pbio.0030328)

Rando Fig S3

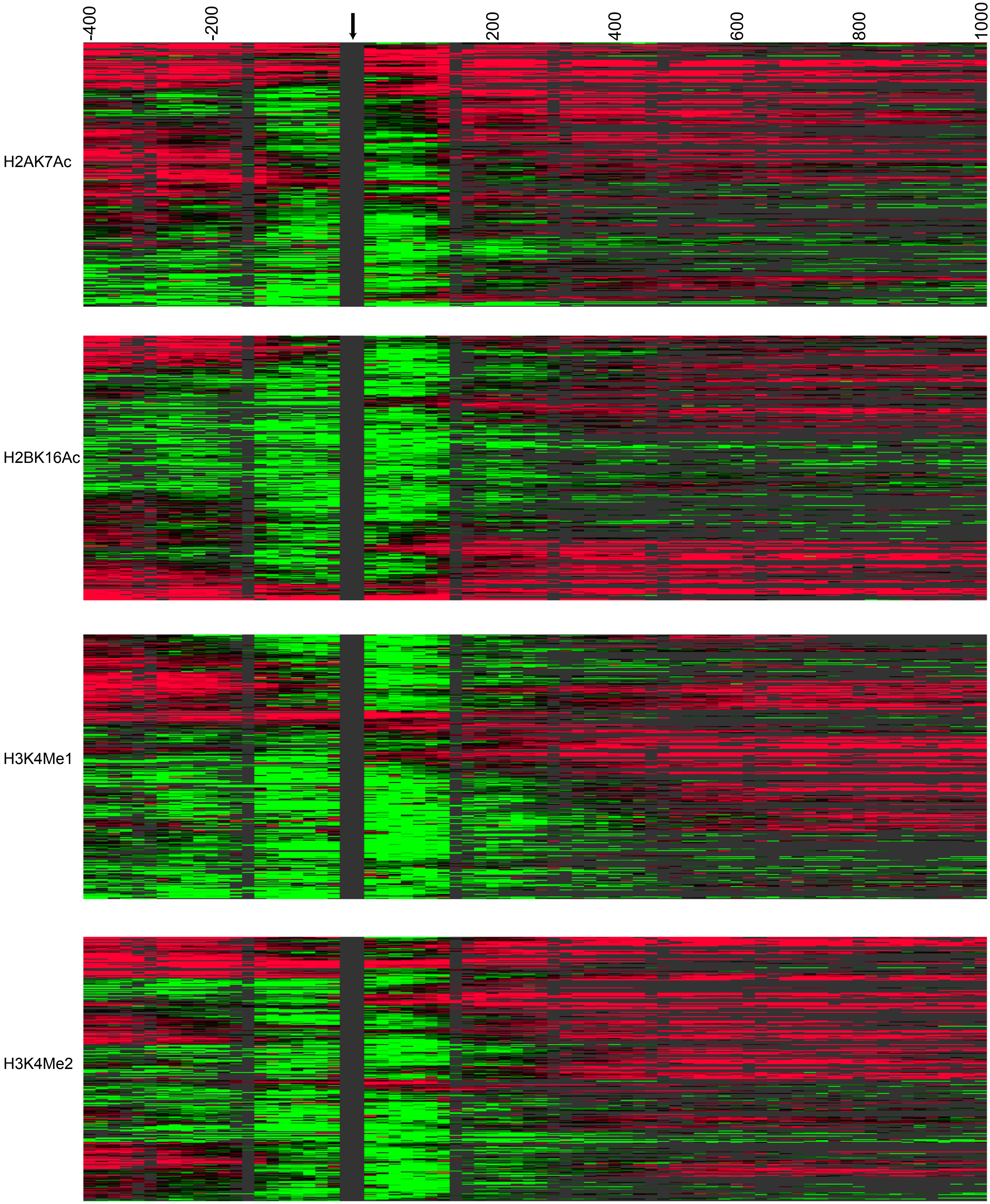
-400
-200
200
400
600
800
1000
H2AK7Ac
H2BK16Ac
H3K4Me1
H3K4Me2

Rando Fig S3

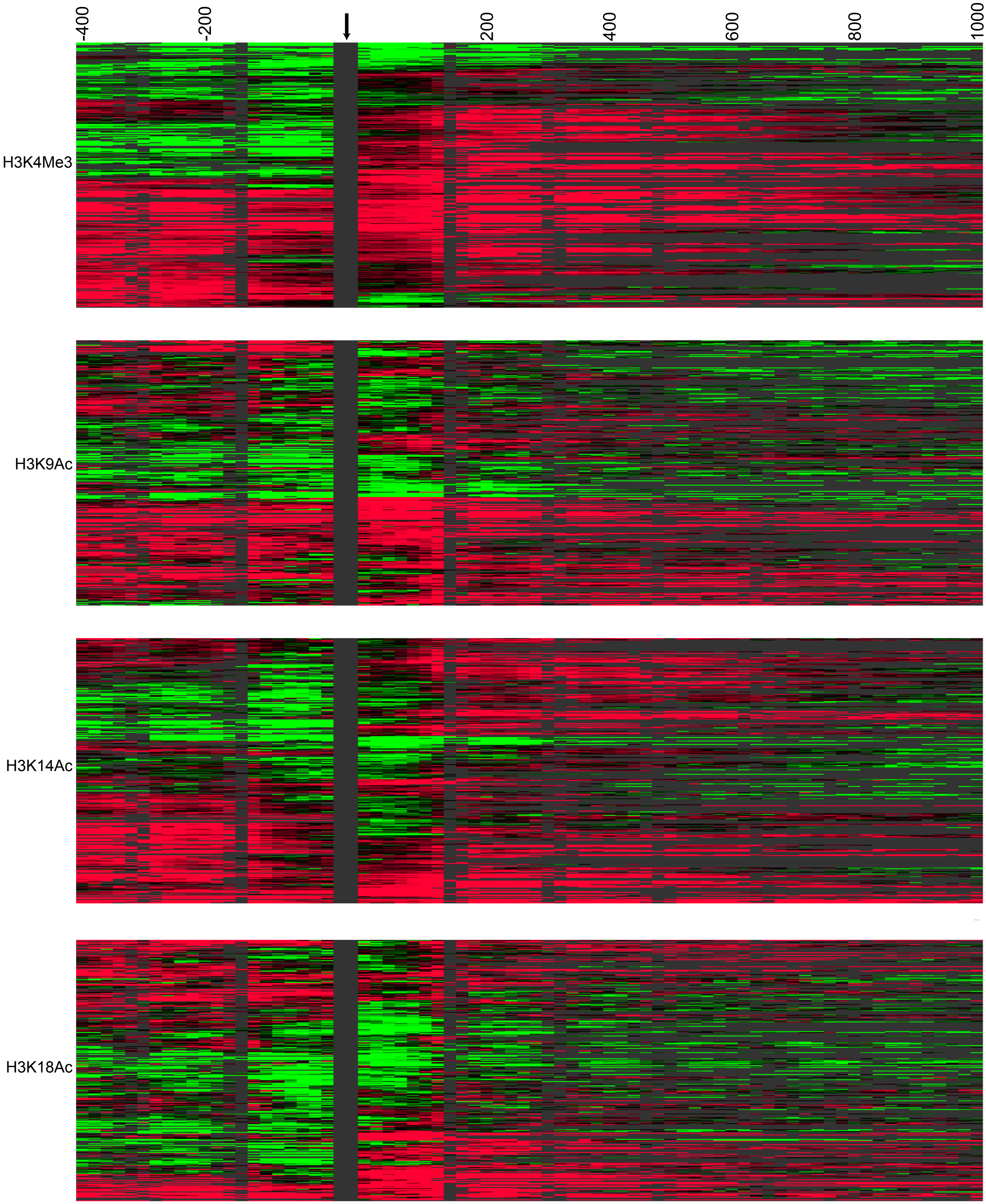

Rando Fig S3

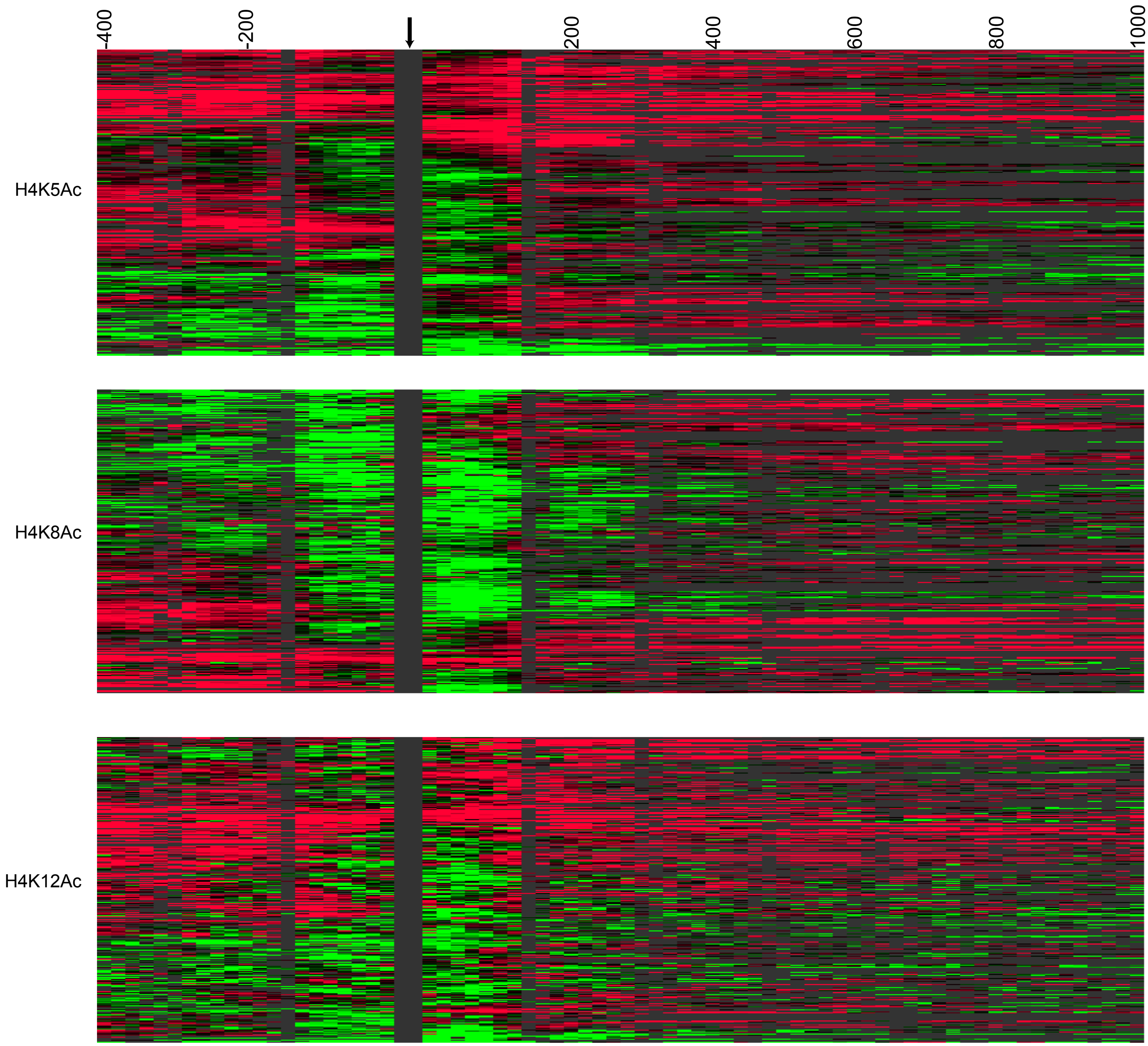

Supplement: Supplementary file 3 [file pbio.0030328.sg003.pdf]
