# Supplementary material for: Single-Nucleosome Mapping of Histone Modifications in S. cerevisiae
Source: PLoS Biol. 2005 Aug 30;3(10):e328. doi: 10.1371/journal.pbio.0030328 (PMC1195719; doi:10.1371/journal.pbio.0030328)
Supplement: Supplementary file 7 [file pbio.0030328.sg007.pdf]

Rando Fig S7

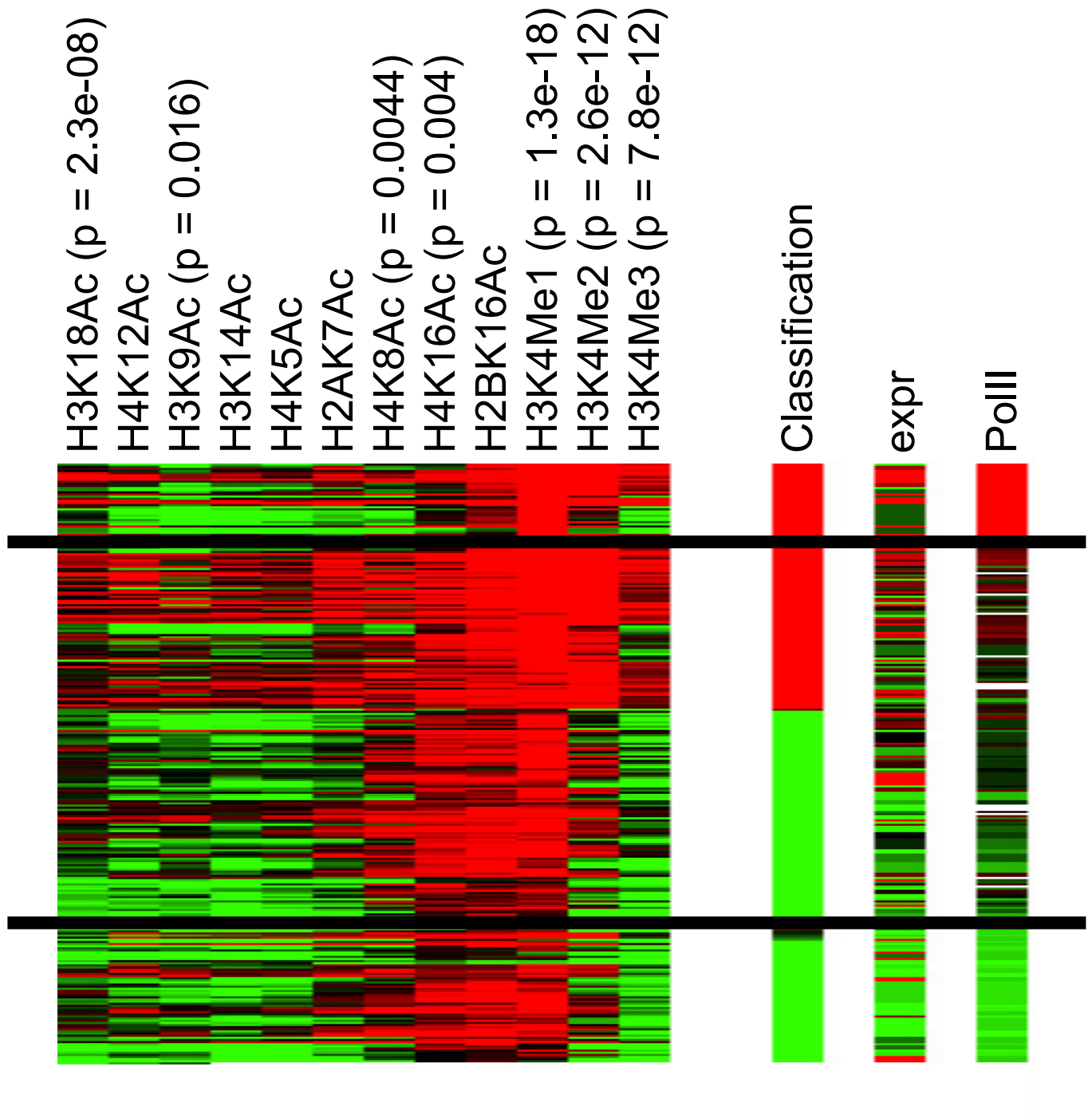

H3K18Ac (p = 2.3e-08)
H4K12Ac
H3K9Ac (p = 0.016)
H3K14Ac
H4K5Ac
H2AK7Ac
H4K8Ac (p = 0.0044)
H4K16Ac (p = 0.004)
H2BK16Ac
H3K4Me1 (p = 1.3e-18)
H3K4Me2 (p = 2.6e-12)
H3K4Me3 (p = 7.8e-12)
Classification
expr
PolII
